# Biodegradable Polymers in Triboelectric Nanogenerators

**DOI:** 10.3390/polym15010222

**Published:** 2022-12-31

**Authors:** Yajun Mi, Yin Lu, Yalin Shi, Zequan Zhao, Xueqing Wang, Jiajing Meng, Xia Cao, Ning Wang

**Affiliations:** 1 Center for Green Innovation, School of Mathematics and Physics, University of Science and Technology Beijing, Beijing 100083, China; 2 Beijing Institute of Nanoenergy and Nanosystems, Chinese Academy of Sciences, Beijing 100083, China; 3 School of Chemistry and Biological Engineering, University of Science and Technology Beijing, Beijing 100083, China

**Keywords:** biodegradable polymers, triboelectric nanogenerator, energy harvester, self-powered sensing, implantable, bioelectronics

## Abstract

Triboelectric nanogenerators (TENGs) have attracted much attention because they not only efficiently harvest energy from the surrounding environment and living organisms but also serve as multifunctional sensors toward the detection of various chemical and physical stimuli. In particular, biodegradable TENG (BD-TENG) represents an emerging type of self-powered device that can be degraded, either in physiological environments as an implantable power source without the necessity of second surgery for device retrieval, or in the ambient environment to minimize associated environmental pollution. Such TENGs or TNEG-based self-powered devices can find important applications in many scenarios, such as tissue regeneration, drug release, pacemakers, etc. In this review, the recent progress of TENGs developed on the basis of biodegradable polymers is comprehensively summarized. Material strategies and fabrication schemes of biodegradable and self-powered devices are thoroughly introduced according to the classification of plant-degradable polymer, animal-degradable polymer, and synthetic degradable polymer. Finally, current problems, challenges, and potential opportunities for the future development of BD-TENGs are discussed. We hope this work may provide new insights for modulating the design of BD-TNEGs that can be beneficial for both environmental protection and healthcare.

## 1. Introduction

In current era, the rapid growth of the internet of things (IoTs) represented by wearable electronic devices and wireless sensor networks has changed every aspect of our daily life, such as health monitoring, medical treatment, communication, entertainment, transportation, environmental protection, infrastructure monitoring, and security [1,2,3,4]. However, in order to ensure reliable and continuous operation of the sensing nodes, so as to realize the full power of IoTs, there arises an urgent demand for prosperous and sustainable energy harvesting devices which can scavenge energies from the ambient environment or our body and charge energy storage devices in a continuous way [5,6,7,8,9]. Meanwhile, there is also a rising dependence of advanced health monitoring and therapeutic technologies on biocompatibility or biodegradability, where biodegradable electronic components can be degraded and disintegrated into monomeric and oligomeric building blocks, thereby reducing their environmental and physiological impacts [10,11,12,13].

Since the first report of Wang’s team in 2012, TENG has been widely used in green electronic systems, not only for self-powered green electronic systems, but also as an active electronic sensor to detect various chemical and physical inputs [14,15]. By harvesting mechanical energy from the surrounding environment and organisms, it can generate green power for real-time monitoring, such as human/animal body, joint movement, all kinds of vibration, deformation, sound wave, liquid/air flow, wind flow, and even electronic health care monitoring [16,17,18,19,20,21,22]. Since then, many studies have been conducted to improve its electrical performance, sustainability, quality, and adaptability [23,24,25]. Due to the fact that the operation of TNEG is based on the coupled effects of triboelectrification and electrostatic induction, polymers such as polytetrafluoroethylene (PTFE), polyvinylidene fluoride (PVDF), nylon, and other organic–inorganic composites have been quite legitimately explored as triboelectric materials due to their position in the triboelectric polarity series [1,11,26,27,28,29,30]. On the one hand, the wide option of triboelectric materials brings tremendous advantages such as low cost, high efficiency, extensive use, and the possibility of continued ascension. On the other hand, like other non-degradable electronics, TENG also causes environmental problems at the end of its useful life, and will eventually be thrown into a landfill or abandoned in an unprotected dump site [31,32,33,34].

Meanwhile, BD-TENG represents a trend in developing green electronic devices. It can be degraded naturally in our environment or resorbed in the body after completing its work cycle without any adverse long-term effects or secondary surgical removal, thus avoiding environmental pollution and improving our health [35,36]. Presently, tunable electrical output capabilities and degradation features can be achieved by using different biodegradable materials. As a result, BD-TENGs with the advantages of biocompatibility, controllability, and biodegradability are fabricated and will find wide applications in in vivo biomechanical energy harvesting and self-powered sensors [37,38,39,40,41,42].

At present, numerous environmentally friendly biodegradable materials, including cellulose, chitin, and silk fibroin, have been found to have biocompatibility, biodegradability, and triboelectric effects [43,44]. For instance, in 2015, Luca Valentini and colleagues took the initiative to employ biodegradable polymer materials into TENG [45]. The solvent casting approach created a TENG based on a biodegradable sodium alginate/graphene oxide nanocomposite sheet. Zheng et al. reported an implantable BD-TENG in 2016 [46]. Due to the layered structure formed by biodegradable polymers (BDPs) and absorbable metal, the biodegradable polymer materials used are poly (lactic acid glycolic acid) copolymer (PLGA), poly (caprolactone) (PCL), polyvinyl alcohol (PVA), poly (hydroxybutyrate valerate) (PHB/V). These polymers are characterized by low cost, commercial purchase, simple processing, etc. At the same time, the component materials can be selected according to the different ability of the triboelectric layer to gain and lose electrons. As a result, the adjustable output voltage of 10~40 V in vitro can be achieved [40,41,42,43]. BD-TENG can be degraded and reabsorbed after completing its work cycle in animals. What’s more, according to the different degradation rates in vivo, the packaging materials can be changed to make the degradation rate of the whole device controllable. Accordingly, Hyun Jun Kim et al. reported a silk-based Bio-TENG in the same year [47]. Since 2017, many studies on BD-TENG from plants have steadily been carried out. So far, BD-TENG has been studied from multiple perspectives including energy harvesting, signal detection, medical care, and other procedures, among which more BDPs have been utilized [31].

**Figure 1:**
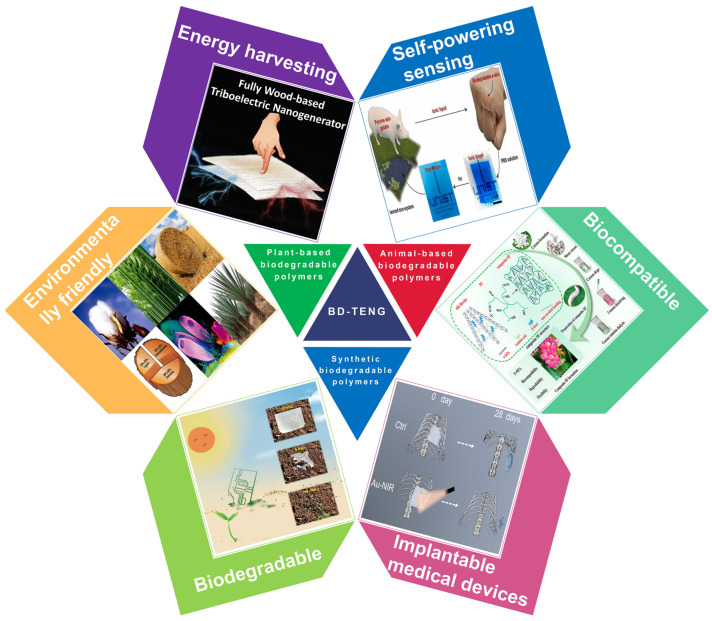
The schematic diagram shows the material sources (plant based, animal based, synthetic) and potential applications (energy collection, self-powered sensing, implantable medical devices) of BDPs proposed in this review in BD-TENG. BD-TENG needs to combine biodegradability, biocompatibility and environmental friendliness.

This review covers the recent research progress of BD-TENG made of biodegradable and biocompatible naturally derived biomaterials. Firstly, the basic principle of TENG and four basic operating modes are introduced. Afterward, we divide the whole of BDPs into three categories and present the recent research progress of BD-TENGs. For convenience, BDPs are divided into three categories according to different sources: plant-based BDPs, animal-based BDPs, and synthetic BDPs. Plant-based BDPs are degradable materials that are extracted from plant sources, such as leaves, wood and cellulose molecules, alginate, rice paper, etc. Similarly, animal-based degradable materials refer to BDPs that are from animal sources, such as chitosan, silk fibroin, and gelatin. Synthetic BDPs are biodegradable substances processed in industry, such as PLA (polylactic acid), PHB/V (polyhydroxy butyrate valerate), PCL (polycaprolactone), etc. With the utilization of these green energy materials, energy harvesting technologies have significantly changed in vitro/in vivo biomedical science and made breakthroughs in the pollution-free reuse of biological wastes (Figure 1). Finally, the future development prospects and challenges of BD-TENG are discussed.

## 2. Basic Principle and Working Modes of TENG

**Figure 2:**
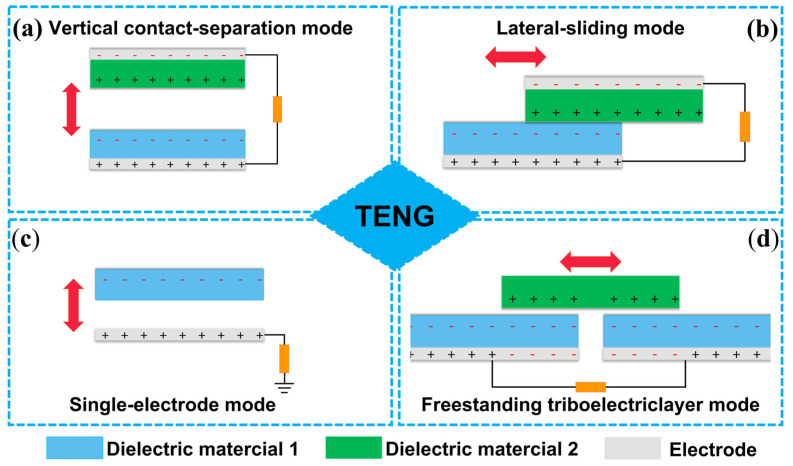
The four fundamental modes of TENG: (**a**) vertical contact-separation mode; (**b**) lateral-sliding mode; (**c**) single-electrode mode; and (**d**) freestanding triboelectric layer mode.

TENG was invented for harvesting mechanical energy on the base of the coupling of friction electrification and electrostatic induction [48,49]. In the triboelectric series, the relative position of two triboelectric layers determines the polarity of their surface electrostatic charges. TENG is divided into four different types according to the operation mode. There are four different modes: (1) vertical-contact separation mode, (2) lateral sliding mode, (3) single-electrode mode, and (4) freestanding triboelectric layer mode. (Figure 2) The electrodes and triboelectric layers of the four modes are arranged differently. The contact and separation of the triboelectric layer will change to generate electrostatic surface charges [50].

The first mode of TENG development is vertical contact separation (Figure 2a). It consists of two different materials with different triboelectric surface potentials. The triboelectric layers come in touch with one another when an external mechanical force is applied, and this causes the triboelectric layer to produce a surface charge. Potential differences occur due to the separation of the two triboelectric surfaces when the mechanical force is released. Electrostatic induction makes an electric charge on the metal electrode attached to the triboelectric layer’s outer surface. To shield the potential difference created by the free charge carrier, the carrier passes over the electrode, producing a pulse of current [50,51]. The harvester is positioned on the triboelectric layer’s outer surface, and it is made up of two triboelectric layers that are vertically separated. This model has been investigated the most because of its straightforward structure [52,53].

The second is the lateral sliding mode (Figure 2b), which is due to the relative sliding of two triboelectric layers and generates triboelectric surface charges. Sliding can be caused by various mechanical motions, including disk and cylinder rotation, plane motion, and more. Different grating structures have been created for transverse sliding mode TENG with outstanding energy harvesting capability. TENG can also produce energy during stretching and be employed successfully in this mode [54,55].

The third is a single electrode mode consisting of a metal or a triboelectric layer grounded through a load resistor (Figure 2c). This model is independent and unrestricted, where any object in contact with triboelectric materials, including human hands can generate triboelectric surface charges. Besides, the two triboelectric layers can be separated without the use of a spacer. Thanks to the single dielectric layer and electrode-only structure, the deformable or self-repairing TENG is simple to realize [56].

The fourth mode is the freestanding triboelectric layer mode, which is composed of freestanding dielectric layers and symmetrical electrodes (Figure 2d). Currents are caused by the contact and subsequent separation of the separate triboelectric layer from the electrode, which results in a potential difference between the two electrodes. The independent TENG can also be divided into contact separation type and sliding type according to the contact mode of the triboelectric layers. Each mode of TENG has its specific benefits and uses [57,58].

## 3. Current Progress in BD-TENGs

### 3.1. BD-TENGs Based Plant Polymers

Recently, the fabrication of TENG has been combined with many plant-based BDPs for environmental protection and biodegradability. Currently, six types of plant degradable materials are used in TENG: paper-based cellulose, bacterial cellulose, leaves, wood, rice paper, plant straw and alginate, etc. [59,60].

Cellulose is one of the most common and abundant macromolecular polysaccharides in nature, accounting for more than half of the carbon in the plant kingdom. In plants, it is usually combined with hemicellulose, pectin, and lignin. Cellulose is one of the bottom materials in the triboelectric series, which has better electron loss capacity. Although these materials deteriorate in the presence of an acid, alkali, and specialized enzymes, the rate of deterioration depends on the material’s particular structure, and the specifically altered molecules can provide cellulose with improved physical and chemical characteristics, as well as greater capacity to transmit electrons [61,62]. For instance, alginate is a byproduct of iodine and mannitol extraction from kelp or Sargassum of brown algae [63], which is typically mixed with other components to generate a composite membrane by cross-linking. Furthermore, alginate materials are polysaccharide derivatives with good biocompatible and simple degradation; the presence of ions makes it easy to dissolve in water or other solutions. These features allow TENG’s manageable degeneration.

In addition to the above, rice paper is also an excellent plant-based biodegradable material commonly utilized in TENG, one which can be easily degraded under natural conditions [64]. Starch, a popular edible polysaccharide derived from rice, corn, and other crops, is also the main raw material of BD-TENG. The main components of leaves are cellulose, a small amount of polysaccharide, and protein [65].

#### 3.1.1. BD-TENGs Based on Paper Cellulose and Starch

Paper-based cellulose is an excellent candidate material for environmentally friendly BD-TENG production because of its good biocompatibility, biodegradability, and recyclability. Biocompatibility is critical for implantation of wearable TENG to prevent rejection, pain, skin irritation, or inflammation [12,61]. Since TENG is biodegradable, it can be dissolved in the human body or in the environment with fewer side effects [66]. In addition, cellulose nanofibers (CNF) have special properties of transparency and high mechanical strength after special treatment, which provides a variety of possibilities for the design of BD-TENG [67]. Up to date, numerous cellulose paper-based TENGs have been produced on the basis of the material’s outstanding flexibility, portability, biodegradability, and reusability, where cellulose paper can be used as an electrode, triboelectric layer, substrate, or perhaps all three materials of TENG at the same time. By modifying cellulose paper, for example, increasing surface roughness and introducing functional groups, the output performance of paper based TENG can be significantly improved. By integrating cellulose paper-based TENG with other types of energy collectors (such as electromagnetic generators and solar panels), the hybrid nano generators can simultaneously collect multiple types of energy. In practical applications, cellulose paper-based TENG can be used as a sustainable power source for self-powered anti-corrosion and antifouling, self-powered electrochemical reaction and droplet controllable operation. It can also be used as an active sensor for pressure sensing, motion monitoring, sound recording and human-computer interaction.

**Figure 3:**
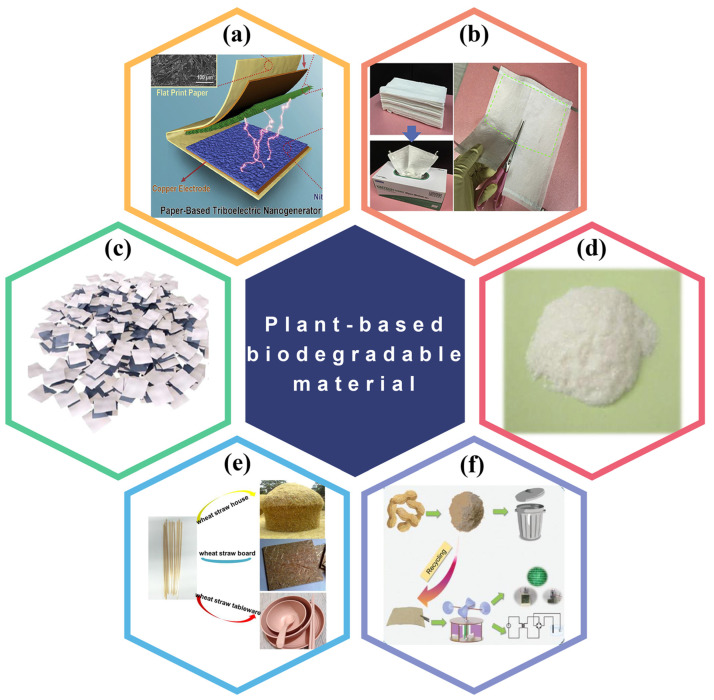
Plant-based biodegradable materials used in BD-TENG. (**a**) A TENG based on CCP and NCM. Reproduced with permission [68]. Copyright 2019, Elsevier Ltd. (**b**) Conductive paper made of commercial tissue paper. Reproduced with permission [40], Copyright 2017, Elsevier Ltd. (**c**) Waste paper used in BD-TENG. Reproduced with permission [69], Copyright 2021, Elsevier Ltd. (**d**) Rice paper powder used in BD-TENG. Reproduced with permission [64], Copyright 2019, Elsevier B.V. (**e**) Wheat straw used as raw material for BD-TENG. Reproduced with permission [70], Copyright 2021, Elsevier Ltd. (**f**) Peanut shell powder used in BD-TENG. Reproduced with permission [71], Copyright 2022, Wiley & Sons, Ltd.

For example, Chen et al. developed a paper-based TENG (PTENG) using widely available cellulose derivatives [68]. The substrate of PTENG is made of printing paper, while the triboelectric layer of PTENG is made of crepe cellulose paper (CCP) and nitrocellulose membrane (NCM) (Figure 3a). This simple and economical method is essential to fabricate environmentally friendly electrical equipment. Wu et al. used a simple and scalable manufacturing process to obtain a PTENG with low cost and high mechanical energy harvesting capability (Figure 3b) [40]. These PTENGs are of great significance for developing green and portable energy-harvesting equipment.

Starch is a widely obtained simple, renewable, eatable, and easily-degrading polymer. Its significant presence of amorphous regions in the form of films and a large number of -OH groups can provide a suitable matrix for cation and ion dissolution, resulting in enhanced dielectric properties in TENGs. Recently, rice paper has been considered as a viable component for constructing biodegradable substrates because it is inexpensive and generally commercially available, compared to other alternatives (such as polymers). Developing high-performance TENG using recyclable rice paper’s has been widely researched. For example, Chi et al. suggested a new BD-TENG that operates on a single electrode [64]. As shown in Figure 3d, Rice paper and transparent conductive ink, both biodegradable materials, are employed as triboelectric materials and conductive electrodes, respectively.

#### 3.1.2. BD-TENGs Based on Micro/Nano Cellulose

Microcrystalline cellulose (MCC), cellulose nanofibers (CNF), and cellulose nanocrystals (CNC) are all types of micro/nano cellulose. The functional materials derived from cellulose have outstanding mechanical characteristics, perfect micro/nano surface roughness, and are lightweight [72]. For instance, Wang et al. developed a water-soluble TENG (WS-TENG) utilizing biodegradable recycled paper and a water-soluble graphite electrode [69]. CNC and methylcellulose (MC) are combined to form CNC/MC films, which are utilized as positive triboelectric materials, and regular MC films are as negative triboelectric materials (Figure 3c). This low-cost, lightweight, BD-WS-TENG can be directly washed away by water and developed as a bandage sensor.

#### 3.1.3. BD-TENGs Based on Crop Wastes and Edible Materials

**Figure 4:**
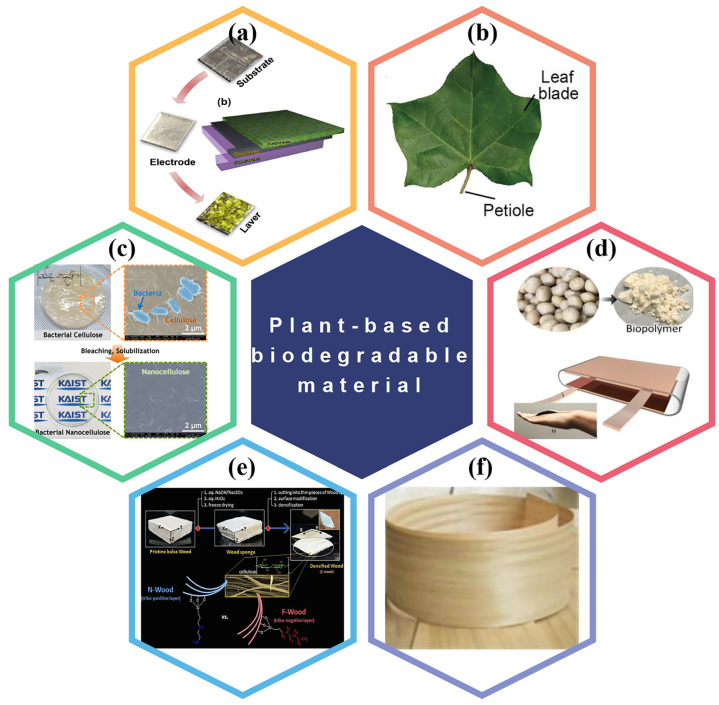
Plant-based biodegradable polymers used in BD-TENG. (**a**) All edible materials derived BD-TENG. Reproduced with permission [73], Copyright 2019, Elsevier Ltd. (**b**) Natural plant leaves used as triboelectric materials. Reproduced with permission [74], Copyright 2020, American Chemical Society. (**c**) Bacterial cellulose used as triboelectric material. Reproduced with permission [75], Copyright 2017, Elsevier Ltd. (**d**) Soybean protein and lignin used as triboelectric materials. Reproduced with permission [76], American Chemical Society. (**e**,**f**) Natural wood used as triboelectric material. Reproduced with permission [77], Copyright 2021, Elsevier B.V. Reproduced with permission [78], Copyright 2022, American Chemical Society.

As a renewable and readily biodegradable environmental protection material, plant straw shows excellent potential in replacing the traditional triboelectric layer. Wheat straw is commonly utilized as natural green manure because of its excellent degradability. For instance, Ma et al. structured a wheat straw natural WS-TENG that can switch from mechanical energy to electrical energy (Figure 3e) [70]. Similarly, peanut shell is a common crop waste generally treated as garbage, and occasionally simply burned in production. As a biodegradable material, however, it has certain advantages in developing BD-TENG due to its rich cellulose and lignin (Figure 3f) [71]. Meanwhile, using edible food materials to develop BD-TENG is also a meaningful research direction. For example, Khandelwal et al. designed a light edible TENG (E-TENG) [73]. The active layer is laver covered with edible silver leaves, the base is rice flakes, and the electrode is edible silver leaves (usually used for candy and oriental food) (Figure 4a). It can be quickly digested in phosphate-buffered saline (PBS) and gastric juice. It is expected to promote the development of edible energy and other electronic equipment such as edible and digestible functional equipment in the future.

#### 3.1.4. BD-TENGs Based on Leaves

Building an intelligent interface between plants and the environment is important for real-time monitoring of plant health while harvesting energy. Harvesting wind energy and raindrops in the environment through plants can be used for various sensors, making agricultural automatic monitoring possible. The leaf is a naturally degradable material utilized in the manufacturing of BD-TENG. For example, Wu et al. reported a green TENG with blade cuticle and internal conductive tissue as the friction material and electrode, and water droplets as the corresponding substance (Figure 4b) [74]. This technology can be combined with urban greening, family gardens, woodlands, and islands for energy harvesting and sensing.

#### 3.1.5. BD-TENGs Based on Bacterial Cellulose

Bacterial cellulose is a porous reticular nanoscale biopolymer synthesized by microbial fermentation, and it is also a polymer with good degradation performance. It has unique biological affinity, biocompatibility, biodegradability, biological adaptability, and no allergic reaction. Therefore, bacterial cellulose shows excellent potential in biodegradable and environmentally friendly triboelectric active materials. For example, Kim et al. were the first to develop a bio-TENG based on bacterial nano-cellulose [75]. The triboelectric layer was made of a transparent, bendable bacterial nano-cellulose sheet. As a positive triboelectric material, the strong electrical feeding capabilities of copper film allows the bacterial nano-cellulose film to generate 4.8 mW m^−2^ of output power (Figure 4c). Zhang et al. built an environmentally friendly and renewable all-cellulose energy harvesting and interaction equipment using bacterial cellulose (BC) and conductive BC [79]. Under the condition of the cellulolytic enzyme, the active substance can be completely degraded within 8 h.

#### 3.1.6. BD-TENGs Based on Lignocellulose

Lignocellulose is an organic flocculent fiber material made from naturally regenerated wood through chemical treatment and mechanical processing [80]. For self-powered equipment such as TNEGs for environmental monitoring and medical rehabilitation, biodegradable environmental protection materials are frequently employed. For example, An et al. developed a BD-TENG based on nanofibers (NF) by using an industrially scalable solution blow-molding process to prepare NF from solutions containing soybean protein and lignin. This TENG based on biopolymers has excellent efficiency in biomedical applications, which makes it a piece of sustainable and environmentally friendly self-powered equipment (Figure 4d) [76]. In addition to being a positive triboelectric material for TENG, lignocellulose has also been developed as a negative triboelectric by adding strong electron absorption groups through modification. For instance, Bang et al. explored an all-wood green TENG [77]. Flexible porous wood triboelectric layers were utilized as positive and negative materials to increase the triboelectric output of TENG. The positive triboelectric layer was treated with trichloro (1H, 1H, 2H, 2H-perfluorooctyl) silane. In contrast, the negative triboelectric layer was treated with N-(2-aminoethyl)-3-aminopropyltrimethoxysilane (Figure 4e). The output voltage of chemically treated TENG was about 20.5 times than that of solemn TENG.

Similarly, Yao et al. proposed a straightforward technique, including pretreatment and post-modification to produce flexible wood triboelectric layer materials with excellent mechanical properties [78]. They attached the positively charged quaternary ammonium group using the cationic modifier 3-chloro-2-hydroxypropyl trimethylammonium chloride (CHPTAC) to the free hydroxyl group on the cellulose backbone. The modified veneer displayed a high surface potential, demonstrating a significant energy difference between the positive and negative triboelectric layers (Figure 4f).

#### 3.1.7. BD-TENGs Based on Alginate and Agar

Sodium alginate is a by-product of extracting iodine and mannitol from kelp or sargassum of brown algae. It is a polysaccharide with good biodegradability and biocompatibility, and also a promising candidate material for BD-TENG [63]. Carrageenan (ϰC) and agar are natural, low-cost edible polysaccharides extracted from red algae and are also used to develop BD-TENG. Kang et al. developed a natural derivative-Carrageenan agar (ϰC-Agar) composite using as high-performance triboelectric material for BD-TENG [81].

#### 3.1.8. Potential Applications of Plant-Based BD-TENGs

##### Plant-Based BD-TENGs for Energy Harvesting

Since the first invention of TENG, obtaining energy from the environment and organisms has always been the most important goal of TENG. The emergence of BD-TENG has brought dawn for providing new environmental protection and degradable energy.

**Figure 5:**
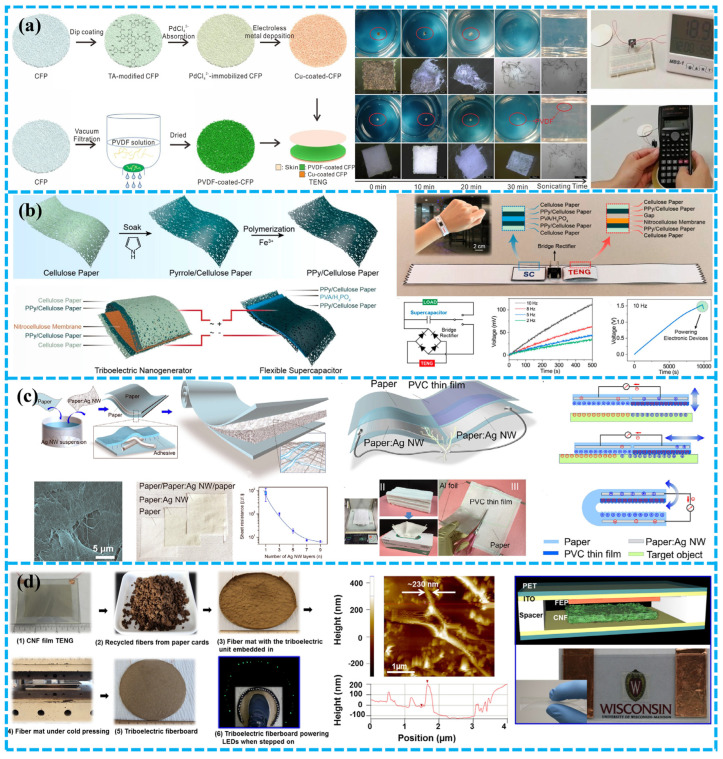
Plant-based BD-TENGs. (**a**) A green, recyclable, and biodegradable CFP-based TENG; A schematic illustration of the fabrication of Cu-coated CFP and PVDF-coated CFP; degradation process of various membranes and the application of TENG based on CFP. Reproduced with permission [82]. Copyright 2022, Elsevier B.V. (**b**) A portable self-charging power supply system integrated with TENG and SC based on cellulose paper and PPy-coated cellulose paper; a schematic diagram of the manufacturing process of PPy coated cellulose paper and the structure diagram of the self-charging power system. Reproduced with permission [83]. Copyright 2020, WILEY-VCH. (**c**) An ultra-soft and cuttable paper-based TENG for mechanical energy harvesting; a schematic illustration of the process used to fabricate the conductive paper; a P-TENG schematic diagram and various working modes; demonstrations of its portability and ability to be tailored. Reproduced with permission [40]. Copyright 2019, American Chemical Society. (**d**) A renewable and BD-CNF-based TENG; manufacturing and performance of CNF-based TENG fiberboard. Reproduced with permission [84], Copyright 2016, Elsevier Ltd. BD-TENG based on rice paper and bacterial cellulose.

Yang et al. built a human-computer interaction and environmentally friendly energy collection system. Cellulose filter paper was used as the triboelectric layer [82]. The unique three-dimensional porous network structure and high crystallinity of cellulose filter paper enable the TENG to have good output performance. Their degradation experiments showed that the prepared CFP-based TENG dispersed and dissolved the composite membrane materials in ultrapure water within 30 min through ultrasonic treatment, which could fully demonstrate its environmentally friendly nature. (Figure 5a). They also deeply investigated the output characteristics of TENGs based on CFP, with a maximum output voltage of 192 V and output current of 9.3 μA. The output power was 736.7 mW m^−2^. The TENG based on CFP can be conveniently used to power commercial electronic products and control computer programs as a wearable interface. This green TENG is of great significance for realizing environmental protection of electronic equipment, promoting energy conservation and emission reduction, and realizing carbon neutralization.

Cellulose paper can be used as an electrode along with a substrate and triboelectric layers. Shi et al. created portable and long-lasting SCPs by combining paper-based TENG with paper-based SC [83]. Cellulose paper showed excellent conductivity after coating with polypyrrole (PPy), The cellulose paper/PPy composite material was then employed as an electrode, cellulose paper was used as the positive triboelectric layer, and nitrocellulose membrane NCM as the negative triboelectric layer (Figure 5b). P-TENG exhibited good triboelectric characteristics (with a V_oc_ of 60 V and a power density of 0.83 W m^−2^). In addition to this, the technology was also developed for applications in self-powered devices. As a flexible, ultralight, and renewable power source, the created SCP can be used to power various electronic devices, demonstrating its potential in flexible and compact green technologies. This research provides essential inspiration for developing degradable TENG made of cellulose paper.

Considering portability, Wu et al. have demonstrated a cutting P-TENG to collect mechanical energy [40]. P-TENG has the advantages of excellent flexibility, lightweight, high conductivity and low cost, and can work normally after being cut into any shape. It can obtain mechanical energy, body movement energy, sound energy and wind energy. By measuring the effectiveness of electronic transmission, the TENG can distinguish many materials, including glass, cotton and wood (Figure 5c).

CNF is a renewable, biodegradable and rich material, and Yao et al. have studied its application in TENG structures, [84]. The flexible transparent CNF film with its nano-structure was used as positive triboelectric material (Figure 5d). When it is trampled by normal people, it can generate up to ~30 V and ~90 μA current to illuminate the 35 LEDs integrated with a power fiberboard which has strong recyclability and excellent mechanical integrity. When it is trampled by normal people, it will generate up to ~30 V and ~90 μA current to illuminate the 35 LEDs integrated with the power fiberboard. This proves the great potential of producing friction electric Bakelite/fiberboard or flooring on a wide range of eco-friendly scales. It can be predicted that CNF and other woods will play an important role in the dynamic development of flooring, packaging and auxiliary infrastructure, as well as the unique ability to efficiently obtain mechanical energy from the environment.

**Figure 6:**
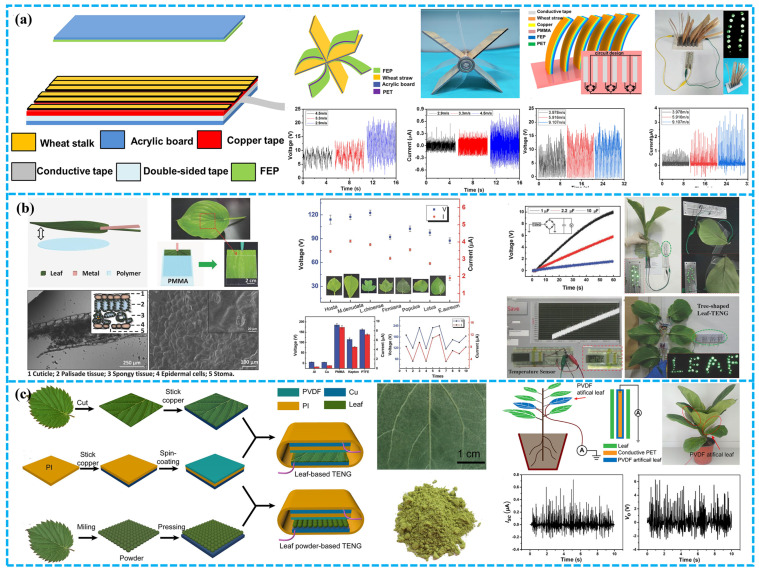
Plant-based BD-TENGs. (**a**) Structure of natural wheat straw assembled TENG; structural diagram of windmill and structural diagram of WS-TENG lawn and various output performance. Reproduced with permission [70], Copyright 2021, Elsevier Ltd. (**b**) A Leaf-TENG assembled from natural leaves for effectively harvesting environmental mechanical energy; photographs of Hosta leaf and Leaf-TENG directly assembled with Hosta leaf and PMMA; photomicrograph of cross section of Hosta leaf, with the inset showing the structure of cross section of common leaves; SEM images of morphology of Hosta leaf; the effect of materials and stability of Leaf-TENG. Reproduced with permission [85], Copyright 2018, WILEY-VCH. (**c**) A blade-based TENG and TENG tree for wind energy harvesting; the schematic illustration of the fabrication process of leaf and leaf powder based TENGs and TENG tree. Reproduced with permission [86], Copyright 2018, Elsevier Ltd.

Ma et al. has structured a wheat straw natural WS-TENG that can switch from mechanical energy to electrical energy [70]. WS-TENG performs well in terms of output and it can charge not just capacitors but also several portable electronic devices such as hygrometers and electronic watches (Figure 6a). More intriguingly, the author built WS-TENG into a windmill and bionic lawn, which can be used as a wind speed sensor. This BD-TENG offers a novel answer for advanced science and technology employed in agricultural production and self-powered sensors used for energy harvesting and application.

Jie et al. presented a green and environmentally friendly innovative blade-assembled Leaf-TENG that can efficiently harvest environmentally and mechanical energy using natural leaves [85]. Under optimal circumstances, the peak values of V_oc_, I_sc_, and maximum output power of natural Leaf-TENG in single electrode mode can reach around 230 V, 9.5 µA, and 45 mW m^−2^ (Figure 6b). Natural Leaf-TENG has been indicated to be competent for powering LEDs and charging capacitors, indicating that it has excellent mechanical energy harvesting capability. The natural Leaf-TENG was constructed autonomously into a tree-shaped energy harvester to gather mechanical energy from the environment over a vast region. Similarly, Feng et al. constructed a TENG using biodegradable natural plant leaves and dry leaf powder to harvest wind energy and obtained high V_oc_ (430, 560 V) and I_sc_ (15, 25 µA) accordingly [86]. To tackle the problem of delicate contact and make maximum use of the leaves, dry leaves are ground into powder (Figure 6c). Poly-L-lysine is utilized to optimize the outputting capability of TENG. Because fresh leaves contain complimentary water, which lowers the electronic generation of natural plant leaves, the performance of dry powder leaf based TENG is better than that of plant leaves. As is shown, the TENG tree consists of both living and artificial foliage, which has promising application potential in distant areas and can be utilized for early warning and indicator lighting. Consequently, plentiful biodegradable materials can be employed to develop future energy.

BD-TENG has also been reported in the application of blue energy; for example, Pang et al. designed a BD-TENG using alginate membrane [63]. It can be used to collect water wave energy. This biodegradable and ecologically responsible TENG based on marine plants have broadened the application field of blue energy.

##### Plant-Based BD-TENGs for Self-Powered Sensing System

TENG can not only be used as an energy harvesting device, but also an excellent sensor in mechanical sensing due to its high sensitivity and short response time. From the perspective of active sensors, TENG can convert mechanical movement into changing electrical signals; the information from a trigger source (amplitude, frequency, position, trajectory, etc.) can be retrieved by analyzing these output signals (voltage, current, frequency, etc.). Environmentally friendly, biocompatible and biodegradable materials also make TENG more unique in the application of transient electronic equipment.

Zhang et al. designed a page marking and anti-theft sensor using commercial printing paper [51]. The printing paper was used as the substrate of the TENG, and then the film made of PET and ITO was placed on the friction surface. This self-powered anti-theft sensor can be inserted into a book. When it contacts the book, the I_sc_ increases from 30 nA to 300 nA with the increase of the grid. When someone gently touches the book, the TENG output voltage of the TENG can light the LED alarm bulb as a warning. More interestingly, TENG can identify matching pages according to different signal amplitudes, accurately determine the number of pages in the book, and record the number of pages turned. These studies are a good starting point for creating simple, portable, flexible and green paper made equipment, as well as the progress of self-powered paper made equipment.

Stephan et al. has developed a CFP-based TENG by dip coating [87]. The CFP-based TENG displayed the V_oc_ and current density of ≈42 V and ≈1 µA cm^−2^, and a power density of ≈25 µW cm^−2^. It can light 40 LED bulbs and charge 0.22 µF capacitors to 8 V within 5 s. The TENG can detect simple human movements, finger tapping, finger friction, and trampling. These results prove that cellulose paper can be employed as the base material for manufacturing superior-behavior BD-TENG.

**Figure 7:**
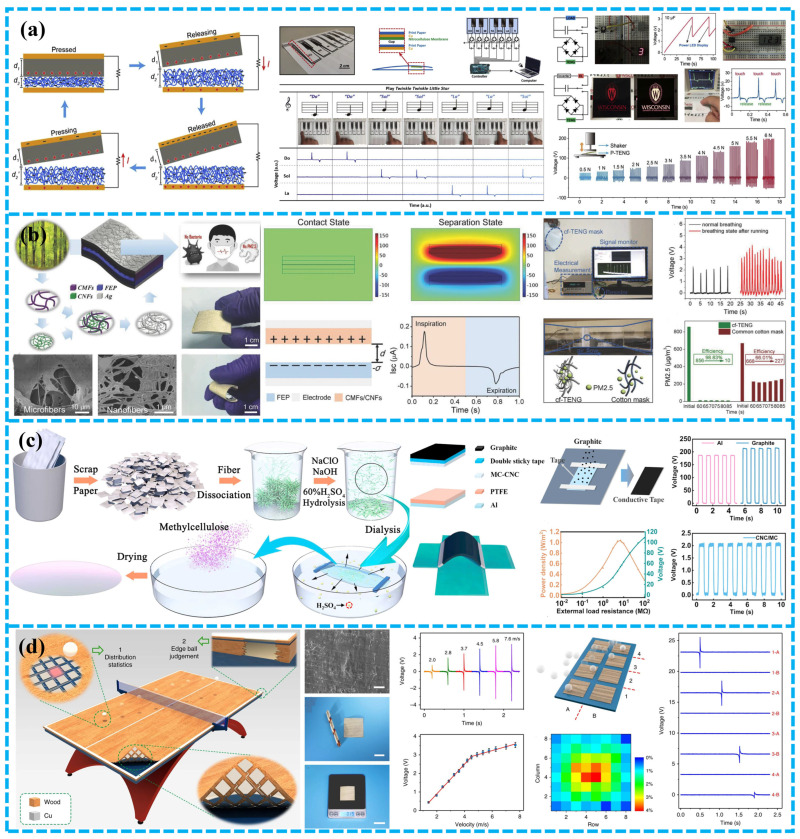
Plant-based BD-TENGs. (**a**) A TENG based on crepe cellulose paper and nitrocellulose film for energy harvesting and self-powered human-machine interaction; a schematic illustration of the P-TENG working principle; a P-TENG-based paper piano for self-powered human–machine interfacing; output voltage of the P-TENG under periodic pressing by a shaker with different applied forces. Reproduced with permission [68]. Copyright 2019, Elsevier Ltd. (**b**) A hierarchically nanostructured cellulose fiber-based TENG for self-powered healthcare products; schematic, microstructure, and photographs of the cf-TENG; working principle of the cf-TENG and Breathing monitoring and PM2.5 removal effect of the cf-TENG. Reproduced with permission [88], WILEY-VCH. (**c**) A recyclable and fully biodegradable water-soluble paper-based TENG; fabrication of CNC from wasted paper and schematic illustration of contact-separation mode TENG between CNC/MC film adhered with graphite electrode and PTFE. Reproduced with permission [69], Copyright 2021, Elsevier Ltd. (**d**) A flexible and durable wood TENG for self-powered sensing in sports big data analysis; fabrication and schematic of the flexible wood-based TENG and smart ping-pong table and application of the W-TENG in a self-powered falling point distribution statistical system. Reproduced with permission [89], Copyright 2019, Springer Nature.

With the development of intelligent electronic devices, human-machine interaction (HMI) technology plays an increasingly important role in daily life. TENG based on cellulose paper can also be employed as an active sensor for HMI because its electrical signal can be utilized as the input signal for operating the machine. Chen et al. reported that a V_oc_ of paper-based PTEN was 196.8 V, I_sc_ was 31.5 µA, power density was 16.1 W m^−2^, and duration exceeded 10,000 times [68]. This PTENG was used as a keyboard for designing and manufacturing paper pianos to achieve automatic human-computer interaction. PTENG can detect human movement, such as finger contact. PTENG array keyboard can realize self-powered real-time interaction with computer (Figure 7a). These studies show the potential of using widely available low-cost cellulose fiber derivatives to produce high-performance paper-based TENG. They also show great potential in HMI, which is critical to creating green and sustainable devices.

Real-time monitoring of human activities and vital signs is critical for health diagnosis and treatment. He et al. presented a cellulose fiber-based self-powered CF-TENG by converting one-dimensional environmentally friendly CMFs/CNFs into two-dimensional CMFs/CNFs/Ag hierarchical nanostructures [88]. The CF-TENG system exhibited an efficiency of 98.83% in removing PM2.5 and can monitor respiratory health without an external power source because of its perfect porous nanostructure and extraordinary power-generating properties (Figure 7b).

Recently, Wang et al. developed a BD-TENG to monitor human physiological signals using CNC and MC [69]. This low-cost, light weight, biodegradable WS-TENG bandage sensor can be directly rinsed with water. According to the change of output voltage, it can accurately distinguish various respiratory states (Figure 7c). This kind of real-time physiological signal monitoring sensor further expands the application of TENG in the medical monitoring field.

Zhu et al. has demonstrated a piece of disposable TENG equipment using environmentally friendly biodegradable starch paper [65]. The materials utilized are both inexpensive and widely available. S-TENG can be made with a simple procedure that combines starch paper and metal wire. This TENG can function as a self-powering human sweat sensor, indicating its potential application in wearable electronic products.

To create a flexible, robust, and high-performance TENG for self-powering sensing in motion big data analysis, Luo et al. used a straightforward and efficient two-step method to prepare wood films with outstanding physical and triboelectric characteristics as the triboelectric materials [89]. Adaptable and long-lasting W-TENG can produce a charge density of 36 C m^−2^, more than 70% greater than natural wood-based TENG (Figure 7d). W-TENG was also employed as an active sensor for intelligent table tennis tables with a speed-sensing system for motion tracking.

Recently, Wang et al. produced a flexible, biodegradable, and flame-resistant FR-TENG using biodegradable black phosphorus and phytic acid as flame retardants. Tannic acid-modified BPNS (TA-BPNS), and PA were added to CNF as non-toxic synergistic flame retardants as the triboelectric layer, and AgNWs were used as the conductive layer. The FR-TENG can be utilized for temperature sensing and as a fire alarm [90]. As a wearable micro/nano power source, FR-TENG can also harness the mechanical energy of human movement to power small electronic gadgets. Being a humidity sensor, the FR-TENG’s output voltage responds linearly to the surrounding humidity. In brief, FR-TENG has a wide range of potential applications in temperature monitoring, fire alarms, and multifunctional wearable electronic devices.

##### Plant-Based BD-TENGs for Implantable Medical Devices

Implantable electronic devices for medical care and health monitoring have advanced quickly. TENG-based biodegradable power supplies have advantages over batteries in preventing issues like secondary medical surgery.

For instance, based on biocompatible medical 317L stainless steel (317L SS) plate and ethyl cellulose (EC) membrane, Wang et al. proposed a healthcare TENG. TENG’s I_sc_ and V_oc_ can reach 245 V and 50 µA under ideal circumstances [91]. After being submerged in simulated bodily fluid for a period of time, the capabilities of the device had not altered appreciably, indicating that it was well-suited for use as a biomedical power source and had good biocompatibility. This shows that cellulose-based BD-TENG has a bright prospect in the application of implantable medical devices.

### 3.2. BD-TENGs Based on Animal Materials

Animal-based BDPs like silk fibroin and gelatin are commonly employed as substrates and encapsulating layers in transitory electronic devices. Recently, animal-based BDPs with special triboelectric effects were used in the BD-TENG and have been widely employed in energy gathering, signal sensing, invasive medicine, and other sectors. Five animal materials-based BDPs are commonly used in TENG: chitosan/chitin, silk and its derivatives, gelatin, and peptides [92,93,94,95,96].

#### 3.2.1. BD-TENGs Based on Chitin and Chitosan

**Figure 8:**
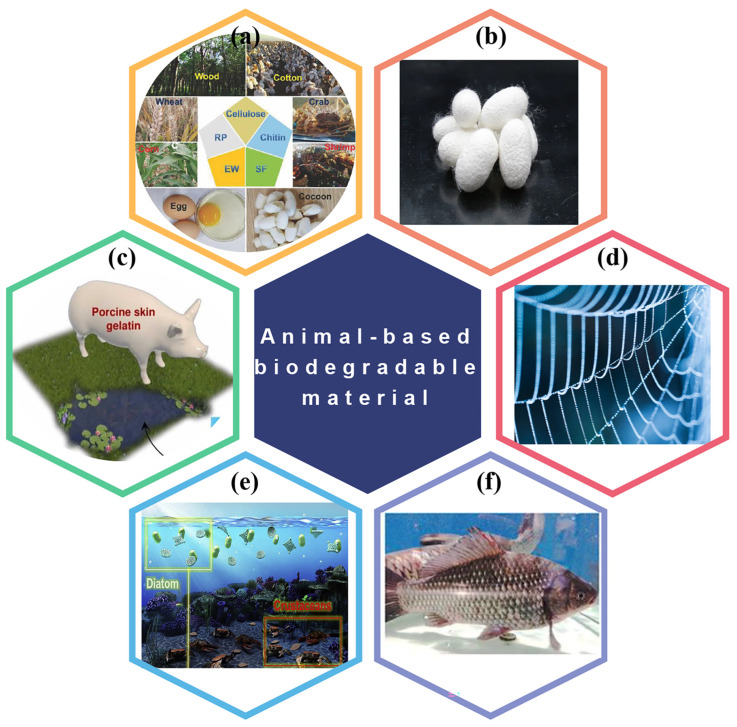
Animal based biodegradable polymers used in TENGs. (**a**,**b**) Natural BDPs originating from nature with wide raw material sources, including silk fibroin, egg, and chitosan. Reproduced with permission [37], Copyright 2021, WILEY-VCH. Reproduced with permission [101], Copyright 2020, Elsevier B.V. (**c**) Gelatin. Reproduced with permission [102], Copyright 2022, Elsevier Ltd. (**d**) Recombinant spider silk fibroin. (**e**) Bio-friendly chitosan. Reproduced with permission [103], Copyright 2020, Elsevier Ltd. (**f**) Fish gelatin. Reproduced with permission [104], Copyright 2020, American Chemical Society.

Hard crustacean (shrimp) and arthropod (insect) shells usually contain chitin and its derivatives (Figure 8e). In terms of electrostatic characteristics, chitin is a naturally occurring polycationic polysaccharide, and electrons can easily be lost. Chitosan, a negatively charged substance, which can be made from chitin, also has the propensity to lose electrons. There are many free hydroxyl and carboxyl groups on the molecular chain of chitosan, which are easy to form hydrogen bonds. Both chitin and chitosan can gradually break down in the body and become little molecules in the environment, so these two materials have strong biocompatibility and absorbability. Moreover, chitin and chitosan exhibit good antimicrobial properties which broadens their potential applications in the biomedical industry [95,97,98].

Chitosan, as a rich natural biopolymer, offers fascinating possibilities in low-cost BD-TENG and other related domains. For example, Wang et al. showed a flexible BD-TENG based on chitosan [99], in which a soft chitosan film as a triboelectric layer can be used for biomedical purposes by placing wearable devices on human skin.

TENG’s safety is also crucial. Kang et al. prepared a BD-TENG by using carboxymethyl chitosan and carboxymethyl cellulose sodium that can be rapidly soluble in water [100]. Additionally, a feeding trial indicated that TENG has edible properties, and even if accidentally ingested, the baby’s health wouldn’t be impacted.

#### 3.2.2. BD-TENGs Based on Silk Fibroin

Silk, egg white, peptides, and other proteins can be employed as the triboelectric layer in TENG. These triboelectric materials exhibit good electron transport properties because of the carboxyl group. These proteins also have strong biocompatibility and can break down quickly in microbial and protease environments. Protein can be easily enhanced and manipulated biologically and chemically, making it more scalable for biomedical purposes (Figure 8a) [37].

Gourray et al. used the AgNW electrode integrated with the cross-linked silk film to monitor human activities and obtain energy from them, which can be used as both a strain sensor and a biological TENG [105]. In 2020, Gong et al. designed a highly transparent, biocompatible and biodegradable biological TENG by adding glycerol and polyurethane (PU) to mesoscopic functionalize regenerated silk fibroin (RSF) for energy harvesting and wireless sensing (Figure 8b) [101]. Jiang et al. produced a totally biodegradable implanted TENG power supply by using natural resources such as rice paper, SF, chitin, cellulose, and egg white to increase the long-term service life of the implantable BD-TENG [37].

#### 3.2.3. BD-TENGs Based on Gelatin

Gelatin generally derives from collagen found in animal connective tissue and gel with incomplete protease hydrolysis. Its attributes include good biocompatibility and biodegradability; accordingly, gelatin is also anticipated to be employed in implanted medical devices [106]. Similar to biological tissues, crosslinked polymer networks in bioderived hydrogels (bio-gel), hold significant promise in developing biodegradable electronic goods with superior mechanical performance. Gelatin-based hydrogels are one of the best options for biodegradable electrical goods due to their cheap price, quick decomposition, and edible qualities. Food-grade gelatin has long been employed in various applications, including tissue engineering, robotics, LEDs, and medicinal sectors (Figure 8c). Additionally, the wide application of gelatin-based hydrogels in clinical trials has proved their biosafety. Thus, gelatin-based hydrogels are regarded as the best biosafety materials for use on electronic skin [107].

For example, Han et al. developed a fish gelatin-based triboelectric nanogenerator (FG-TENG) made of flexible, environmentally benign, and multifunctional FG films (Figure 8f) [104]. FG has a strong electron donating ability and outstanding mechanical durability. Pan et al. reported a fully BD-TENG based on gelatin film and electrospinning polylactic acid nanofiber film, which is biologically and environmentally friendly and will not have adverse effects on the environment or on the human body [108].

#### 3.2.4. Possible Applications of Animal Extracts-Based BD-TENGs

##### BD-TENGs Based on Animal Extracts for Self-Powered Active Sensing System

The advantages of environmental protection, biocompatibility and biodegradability of animal extracts endow BD-TENGs with more unique characteristics in the application of transient electronic equipment, especially in self-powered active sensing.

**Figure 9:**
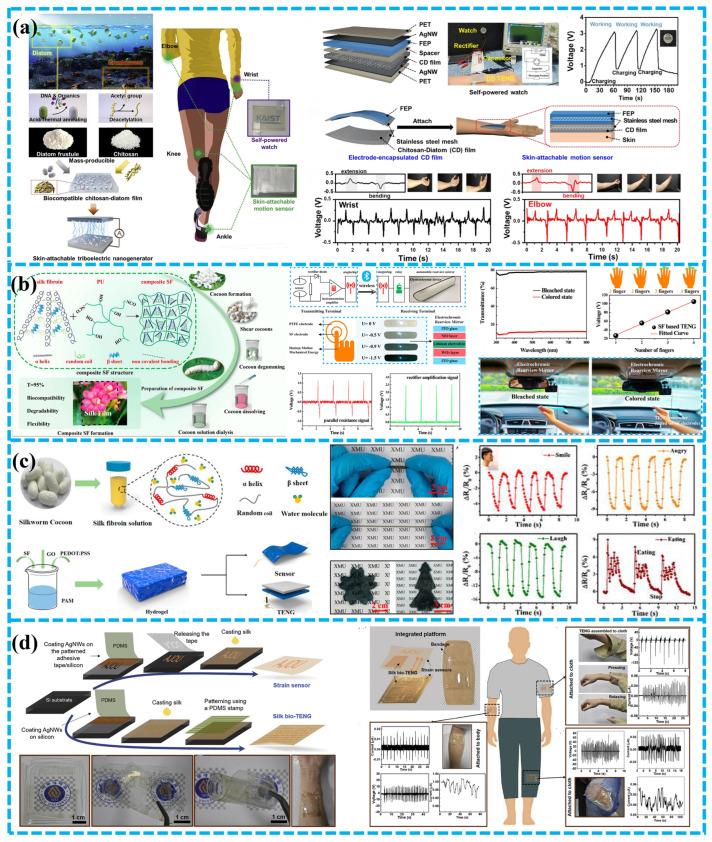
BD-TENGs based on animal extracts for self-powered active sensing system. (**a**) A skin adherent and bio-friendly chitosan diatom-based TENG; synthesis of mass-producible diatom frustule and chitosan from naturally abundant ocean biomaterials and biocompatible chitosan-diatom triboelectric nanogenerator; applications of chitosan-diatom TENGs to wearable devices operated from human motion. Reproduced with permission [103], Copyright 2020, Elsevier Ltd. (**b**) A TENG for SF, which can be stretched, stabilized, and degradable through mesoscopic doping, is used to adjust the color/transmissivity triggered by finger movement and harvest biomechanical energy in vitro or in vivo for intelligent wireless communication. Reproduced with permission [110], Copyright 2021, American Chemical Society. (**c**) A stretchable, biocompatible, and multifunctional SF hydrogel for wearable strain/pressure sensors and TENG; application of the PSGP sensor for monitoring some body signals. Reproduced with permission [111], Copyright 2020, American Chemical Society. (**d**) A skin contact driven single electrode protein TENG and strain sensor for biomechanical energy harvesting and motion sensing; schematic diagram and photograph images of the integrated strain sensor and the bio-TENG on a bandage. Reproduced with permission [105], Copyright 2019, Elsevier Ltd.

To acquire biomechanical energy from human movement, Kim et al. have developed a chitosan diatom composite membrane TENG, which can be used as a wearable device without causing discomfort [103]. The diatom shell has a porous structure, which can be produced on a large scale in the marine environment and can be used as a biocompatible additive. With the addition of diatom shell, the electrical properties of the chitosan membrane were significantly changed and the performance of chitosan diatom-based TENG was 3.7 times higher than that of pure chitosan TENG (Figure 9a). In addition, the author also developed a motion sensor based on biocompatible TENG.

In 2019, Jiang et al. developed an all-electrospinning flexible TENG for monitoring human actions [109]. The negative triboelectric layer of TENG was prepared by an inventive combination of a very negative and highly conductive MXene nanosheet and PVA. In addition, SF was selected as the electron donor of the electrospinning nanofiber membrane due to its favorable triboelectric and biocompatibility characteristics. Excellent strength and stability were obtained in the electrospinning TENG, and the maximum power density of 1087.6 mW m^−2^.

In the previous work, the original SF was generally used as the triboelectric material, which has limited improvement on the performance of TENG. Many studies have started to change SF materials to increase the performance of SF-based TENGs. Xu et al. modified SF membrane by doping and constructed a flexible, elastic, and fully bioabsorbable TENG [110]. Moreover, the TENG can be adhered to the finger to prevent the interference of the rear driving beam and be used to intelligently control electrochromic function of the rear-view mirror. Additionally, SF have excellent physical reliability and chemical stability (resistance to 100 °C temperature and 3–11 pH) and have been used in many fields (Figure 9b). SF can also be employed in vitro or in vivo to gather biomechanical energy.

He and colleagues reported the development of a portable, biodegradable pressure and strain biosensor on silk fibroin hydrogel [111]. The pressure/strain sensor has a broad detection range and can be utilized to track human physiological signs, including breathing, pulse, face posture (Figure 9c), etc. This research demonstrates that silk-based hydrogels have many applications, including TENG, power supplies, soft robotics, and portable digital equipment.

Wearable/attachable electronic devices are essential for a seamless human-machine interface. Gogurla et al. obtained a flexible, transparent and skin/textile compatible TENG and strain sensor using the nanostructured silk protein and AgNWs embedded in the silk nanostructures, which can be used for biomechanical energy collection and motion sensing [105]. It is utilized for motion sensing and the harvesting of biomechanical energy. The device exhibits a very high strain coefficient and can detect joint bending and straightening reliably (Figure 9d). In their work, finger contact can drive the silk biological TENG and generate 2 mW cm^−2^ sufficient to power the LED. The device’s ability to function as a touch sensor on electronic gadgets was realized by optically transparent biological TENG. To track strain and gather biomechanical energy, a silk chip containing a strain sensor and bio-TENG was linked to the skin and fabric.

**Figure 10:**
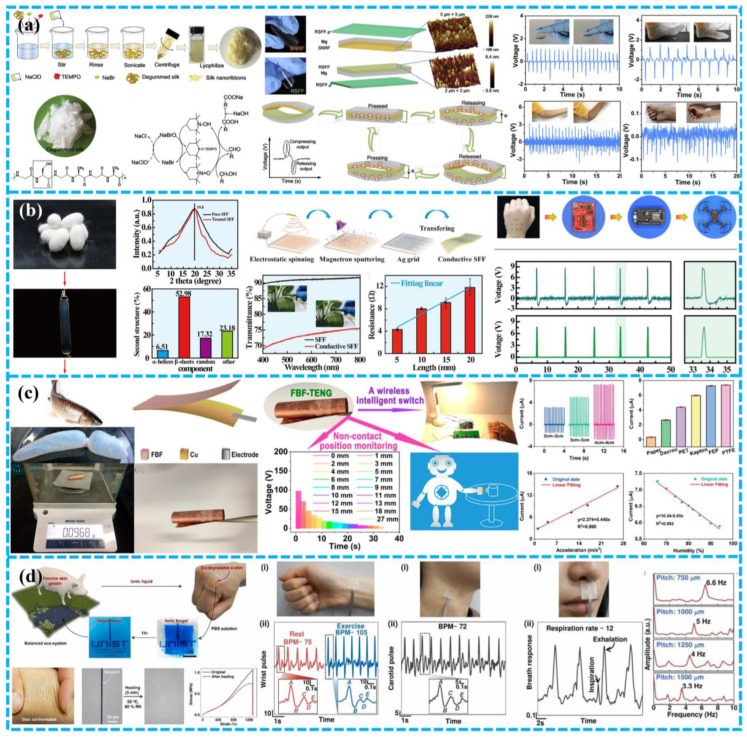
BD-TENGs based on animal extracts for self-powered active sensing system. (**a**) A pulse-driven bio TENG based on silk nanoribbons; a schematic illustration of silk nanoribbon (SNR) preparation process; real-time monitoring of different parts of the body. Reproduced with permission [112], Copyright 2020, Elsevier Ltd. (**b**) A transparent, stretchable, and degradable protein electronic skin for biomechanical energy harvesting and wireless sensing; illustration of the SFF preparation and property characterization; application of tactile sensing based on the SFF-STENG in the internet of things by WLAN. Reproduced with permission [101], Copyright 2020, Elsevier B.V. (**c**) An intelligent electronic skin based on the biocompatibility and biodegradability of flexible single electrode fish bladder film TENG (FBF-TENG). Reproduced with permission [113], Copyright 2021, American Chemical Society. (**d**) A super-stretchable, tough, healable, and biodegradable triboelectric device with microstructure and ion crosslinked bio-gel; schematic of the balanced full cycle of an eco-system with zero waste footprint: manufacturing of the porcine skin gelatin-based ionic bio-gel, utilisation as biodegradable device, degradation of the food colored ionic bio-gel upon disposal in PBS buffer (pH ≈ 7.4) solution at 37 °C temperature; applications of the iSBTENG to low-frequency human vital sign monitoring and high-frequency acoustic wave sensing. Reproduced with permission [102], Copyright 2022, Elsevier Ltd.

Niu et al. constructed a bio-TENG based on silk nanoribbons (SNR), and a novel SNRF and regenerated SF membrane to adjust the biodegradation rate of the apparatus employed [112]. Since it contains just silk and magnesium, it is highly biocompatible, biodegradable, and has a manageable life. With the help of the 2,2,6,6-tetramethyl-1-piperidinyloxy (TEMPO) oxidation system, it is possible to produce natural SNF structures from the silk peeling and obtain SNR with controllable size and steady water conditions (Figure 10a). The maximum voltage, current, and power density of biological TENG were 41.64 V, 0.5 µA, and 86.7 mW m^−2^, respectively. The in vitro testing has proved that the device’s silk and magnesium (Mg) are 100% biodegradable and biocompatible. Furthermore, the TENG exhibited excellent sensitivity for detecting light forces, including finger tapping, foot, elbow, and even human pulse movements.

Self-powered flexible sensors play an increasingly important role in wearable and even implantable electronic devices. In 2020, Gong et al. designed a Bio-TENG that was highly transparent, biocompatible, totally biodegradable, and flexible for energy harvesting and wireless sensing [101]. To begin, glycerol and polyurethane (PU) were combined to produce regenerated silk fibroin (RSF). Next, silver nanowire was created on the silk membrane to generate a thin layer that was breathable, elastic, degradable, adaptable, and this was utilized as an electrode (Figure 10b). The proposed Bio-TENG showed high transparency (83% Ag mesh layer), high extensibility, and it can power portable electronic devices. Furthermore, Bio-TENG might be used as a touch/pressure detecting artificial electronic skin or as an internet of things switch.

Han et al. developed a flexible, environmentally friendly and multi-functional fish glue based TENG, which shows excellent performance and can easily drive 50 commercial LEDs [104]. It is important that the devices made of fish glue can act as self-powered sensors to track physiological signals, including breathing, joint movement, and finger contact.

Similarly, Ma et al. also presented a fish bladder film TENG (FBF-TENG) for brilliant electronic skin that is multipurpose, super flexible, and very sensitive [113]. Natural materials with high biocompatibility and degradability were as the triboelectric layer (Figure 10c). The output current density and charge density of the FBF-TENG for harvesting bioenergy were close to 4.56 mA m^−2^ and 25 C m^−2^. FBF-TENG also possessed outstanding acceleration and humidity sensitivity, with values of around 446 nA s^−2^ m^−1^ and 50 nA/%RH, respectively. Surprisingly, the electronic skin constructed based on FBF-TENG also provided non-contact position-sensing capability in the 0–27 mm range. Its versatility makes it an excellent choice for many applications, including human-computer interface, intelligent interface, artificial limb, robot design, wearable electronic devices, etc.

The degradation rate of biodegradable materials is also critical. Given this, Sujoy Kumar Ghosh and colleagues designed an excellent-performance ionic bio-gel equipment through a 3D microstructure design with a wholly healable and degradable ion-crosslinked bioresource gelatin [102]. The 3D interlocking micro-pattern architecture of thin ion bio-gel has good tensile properties, up to 4000%, resulting in a substantial-high value of 3000% for the working tensile of the produced BD-TENG. The TENG has variable degradability and produces no waste. Furthermore, the TENG can be recovered even after mechanical damage due to its self-healing feature (Figure 10d). Additionally, the TENG based on the ionic biological gel can be utilized for excellent-outcomes electronic skin with good sensitivity and can be applied to human health monitoring, acoustic sensing, roughening up unknown objects, and human-machine interface without having a negative impact on the human body or ecosystem.

##### TENG Based on Animal Extracts for Implantable Medical Devices

Implantable medical devices can monitor necessary physiological signals and electrical stimulation to help cure diseases and provide better patient care. But at the end of their useful lives, most implanted medical devices must be eliminated or displaced by complicated invasive surgery. Electronic devices that can be absorbed or dissolved by the body, such as implantable, biodegradable technology, are a new trend aiming to prevent invasive surgery [114].

**Figure 11:**
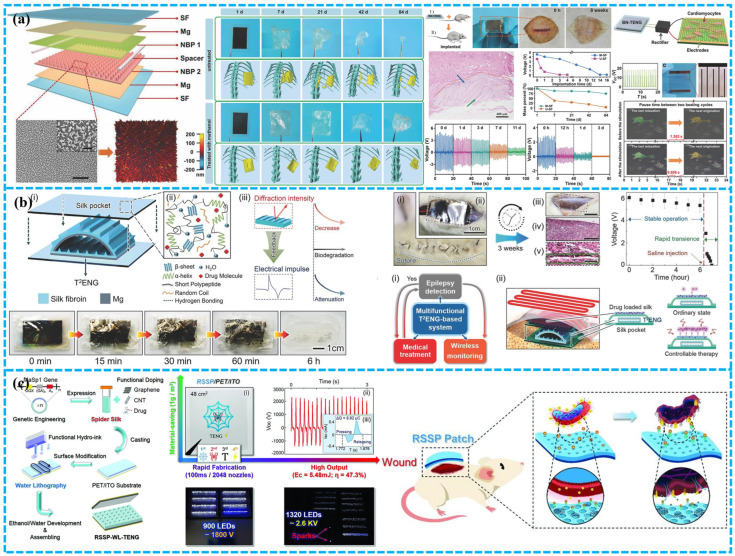
TENG based on animal extracts for implantable medical devices. (**a**) A full BD-TENG based on natural materials. Reproduced with permission [37], Copyright 2021, WILEY-VCH. (**b**) An implantable, biodegradable, and multifunctional system based on silk, self-powered by transient TENG (T^2^ENGs). Reproduced with permission [39], Copyright 2018, WILEY-VCH. (**c**) A biocompatible TENG with programmable triboelectric properties, multi-function, large-scale manufacturing capability, and excellent output performance through genetically engineered RSSP. Reproduced with permission [115], Copyright 2018, WILEY-VCH.

In 2018, Jiang et al. produced a totally biodegradable implanted TENG power supply out of natural resources such as rice paper, SF, chitin, cellulose, and egg white to increase the long-term service life of the implantable BD-TENG. This equipment can produce up to 21.6 mW m^−2^ of electricity at its highest efficiency (Figure 11a) [37]. The device can be completely biodegraded and reabsorbed in SD rats. By modifying the performance of the packing film made of silk fibroin, the working life of TENG can be changed from a few days to a few weeks.

For real-time in vivo seizure monitoring and treatment, Zhang et al. developed biodegradable self-powered implantable transient T^2^ENGs made of SF and absorbable metals (Mg) [39]. Mg is utilized as an electrode and a negative triboelectric substance. Once the device was subcutaneously implanted in the rodents, the movement of SD rats would drive the motion of the silk layer and Mg layer, turning biomechanical energy into electrical energy (Figure 11b). The power supply can be utilized for monitoring epilepsy according to various intensity frequencies and amplitudes that correlate to different health statuses. A completely automated biomedical multi-function system that incorporates T^2^ENGs for real-time in vivo monitoring and treatment of seizures was also presented by the author to accomplish the integration of illness diagnosis and real-time therapy. The entire system comprises a wireless transmitter, signal amplifier, and implanted T^2^ENG sensor with phenobarbital-equipped epileptic treatment molecules. When the sensor signal rises beyond the predetermined threshold, epilepsy can be diagnosed. The thermal unit also initiates medicine administration. Epileptic induction signals appeared after the injection of penicillin G sodium (PNG) solution for 30 min. The system of medical care will be activated whenever epileptic signs are discovered. After 10 min of therapy, the experimental rats got significant improvement from their seizures. However, it should be emphasized that the rat’s mobility, such as running and leaping, as well as the mechanical stimulation of the surrounding environment, would significantly impede the measurement and treatment operations.

Zhang et al. developed a drug-free “antibacterial patch” of TENG made with genetically modified recombinant spider silk protein (RSSP) to promote functional diversity [115]. To alter the charge affinity and enhance mechanical strength, the RSSP gene sequence was created and expressed in Escherichia coli. This is because an enormous molecular weight will produce fewer chain ends, resulting in higher mechanical strength and uniformity. Purified RSSP aqueous solutions were combined with chemical and biological fillers, such as carbon nanotubes, and therapeutic molecules to provide varied functionalization (Figure 11c). To further improve triboelectric properties, the author additionally integrated functional particles/molecules and created ridge patterns on the RSSP surface using ink-jet-based horizontal lithography. Staphylococcus aureus inhibition is one example of a drug-free RSSP patch with outstanding antibacterial activities in vivo. These investigations have demonstrated the viability of implanted BD-TENG for upcoming intelligent multifunctional biomedical systems.

### 3.3. BD-TENGs Based on Synthetic Biodegradable Materials

**Figure 12:**
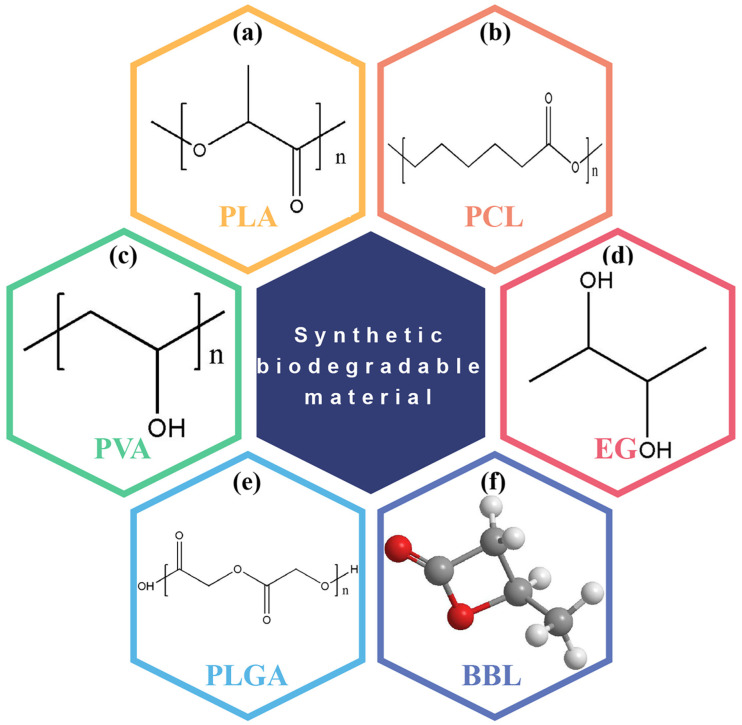
Synthetic degradable material used in TENGs. (**a**) PLA, polylactic acid; (**b**) PCL, polycaprolactone; (**c**) PVA, polyvinyl alcohol; (**d**) EG, ethylene glycol; (**e**) PLGA, poly(lactic-co-glycolic acid); and (**f**) BBL, β-butyrolactone.

Currently, polylactic acid (PLA), polylactic acid glycolic acid copolymer (PLGA), polyvinyl alcohol (PVA), polycaprolactone (PCL), and poly 3-hydroxybutyrate-co-3-hydroxyvalerate (PHB/V) are the five types of artificially degradable materials utilized in TENG (Figure 12) [60,116,117].

#### 3.3.1. BD-TENG Based on PLA and PLGA

As a brand-new synthetic material, PLA excels in material qualities and is regarded as one of the best alternatives among polymer materials derived from petroleum. Additionally, the breakdown product of the ester bond polymerization of PLA and PLGA is a kind of human metabolite [118]. These materials can thus be employed effectively in implantable applications due to their bioabsorbable qualities.

#### 3.3.2. BD-TENG Based on PCL and PVA

PCL and PVA are both petroleum-derived biodegradable materials. Ethyl acetate is typically utilized as the monomer of PVA because vinyl alcohol is not remarkably stable. PVA film has strong mechanical, biodegradable, and biocompatible qualities but has weak thermal stability [119,120,121,122,123]. Typically, PCL has a high total molecular weight and a relatively slow rate of biodegradation compared to other polymer compounds [124].

#### 3.3.3. BD-TENG Based on PHB/V

PHB/V is a polymer made from PHB that is a hydroxybutyric acid and valeric acid copolymer [125]. Chemical processes are primarily used in the production of PHB/V. PHB/V-based materials have strong mechanical characteristics and are biocompatible and biodegradable like the other materials discussed.

#### 3.3.4. Possible Applications of BD-TENGs That Are Developed on Synthetic BDPs

##### TENGs That Are Based on Synthetic BDPs for Energy Harvesting

**Figure 13:**
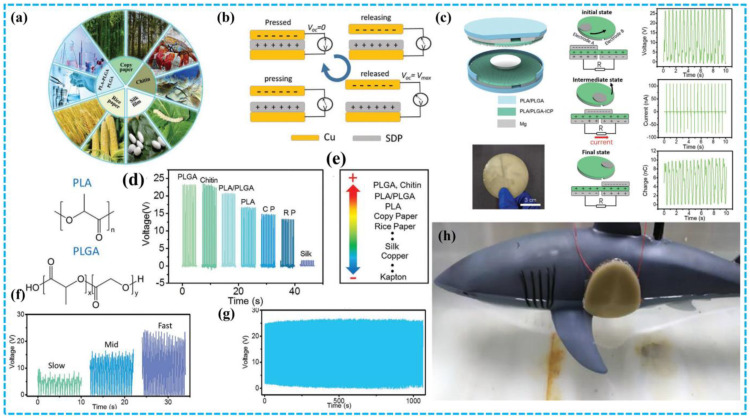
TENG based on synthetic degradable polymer for blue energy harvesting: (**a**) triboelectrification properties of biodegradable materials, and biodegradable materials from different sources; (**b**) the scheme of the working mechanism of a contact-separation TENG; (**c**) a structure diagram, photograph, and working mechanism of the SD-TENG and V_oc_, I_sc_, Q_sc_.; (**d**) the open-circuit voltage of the TENG using different SDP materials; (**e**) a brief tribo-series of biodegradable materials; (**f**) output voltage of SD-TENG at different vibration speeds; (**g**) the stability of the SD-TENG for about 1000 cycles; and (**h**) a photograph of a SD-TENG tested in water. Reproduced with permission [126], Copyright 2020, WILEY-VCH.

BD-TENG developed by using synthetic BDPs has been reported many times. For example, Chen and colleagues investigated the triboelectric characteristics of the synthetic polymers PLA, PLGA, and PLA/PLGA blends [126]. PLGA, which is susceptible to saltwater degradation and PLA, which isn’t susceptible to seawater degradation, both disintegrate slowly in seawater and totally degrade in 9 months (Figure 13). PLA/PLGA has been discovered to have outstanding electrification performance, superior triboelectric performance, and mechanical strength. In their work, the author developed a hollow plate seawater degradable TENG (SD-TENG) using PLA/PLGA as the charge layer and Mg as the electrode, which proved the capacity to transfer the vibration energy of water waves into electrical power. This work demonstrates a clean approach to utilizing wave energy to offer electricity for future temporary maritime equipment without producing non-degradable marine garbage.

##### TENGs Based on Synthetic Degradable Polymers for Self-Powered Sensing System

**Figure 14:**
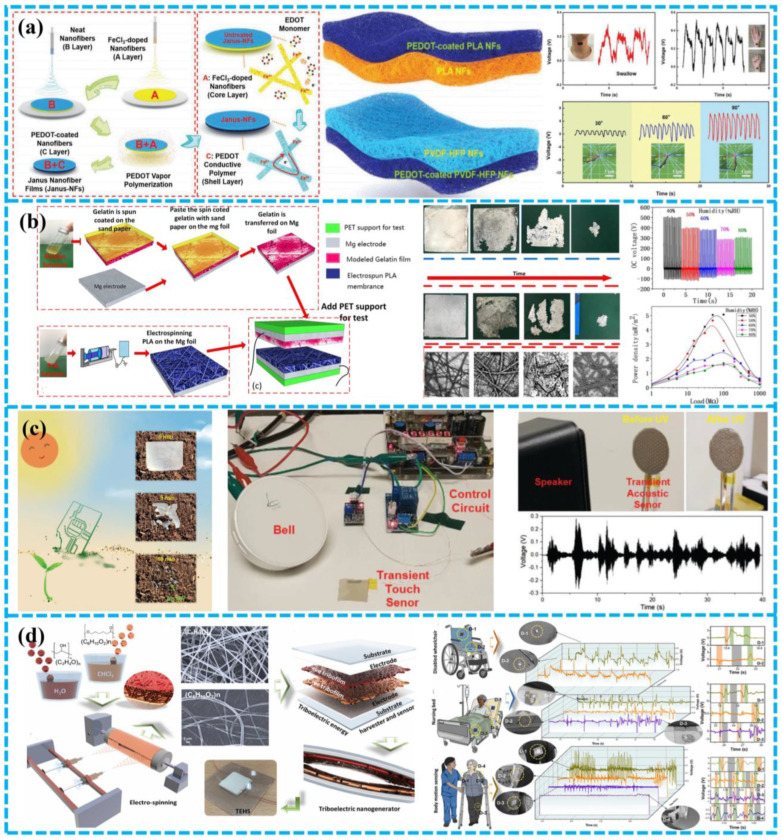
TENGs based on synthetic degradable polymers for self-powered sensing system. (**a**) A TENG based on crepe cellulose paper and nitrocellulose film for energy harvesting and self-powered human-machine interaction. Reproduced with permission [127], WILEY-VCH. (**b**) A full BD-TENG based on electrospinning polylactic acid and nanostructured gelatin film. Reproduced with permission [108], Copyright 2017, Elsevier Ltd. (**c**) A photothermal tunable biodegradable implantable TENG for tissue repair. Reproduced with permission [128], Copyright 2018, Elsevier Ltd. (**d**) A fully biocompatible and biodegradable triboelectric fiber membrane for triboelectric energy harvesters and sensors (THES). Reproduced with permission [129], Copyright 2022, Elsevier Ltd.

Qin et al. developed a porous structure TENG based on nanofibers through electrospinning PLA to meet the human body’s demand for comfort in wearable technology [127]. The uses electrospinning nanofiber film is highly flexible, extremely light, and has excellent air permeability. At the same time, it can capture the energy of body motion and enable real-time monitoring of throat vibration and wrist grabbing (Figure 14a).

Good water solubility of the equipment indicates that it is sensitive to humidity, but this leads to poor stability of the equipment. Pan et al. suggested a BD-TENG based on gelatin film and electrospinning polylactic acid nanofiber film to increase the mechanical reliability and stability of the biomaterial-based TENG device [108]. The output performance was improved with a value of 500 V (Figure 14b). After up to 15,000 contact cycles, BD-TENG exhibited exceptional mechanical stability and dependability. The biodegradation experiment demonstrated that all materials in this TENG can be entirely broken down in the water in around 40 days.

In order to better control the lifetime of devices and expand the application scenarios of transient electronic devices, researchers have proposed using metastable polymers as an alternative method to realize device transients. Previously reported transient energy harvesters employing biodegradable polymers need to be based on solution degradation, and the applications in non-biological situations are restricted. In light of this, Wu et al. designed a short-term, sunlight-activated degradable TENG. The primary substrates were acid-sensitive polyphenylene formaldehyde (PPHA), photoacid generator (PAG), and photosensitizer (PS) [128]. In the sunshine of winter, the constructed equipment can degrade quickly. The pace of degradation can be controlled further by varying the quantity of photosensitizer (Figure 14c). This research not only expands the application area of TENG as a transient power supply and sensor but also extends the usage of transient functional polymers to advanced energy and sensing applications.

Santayana Adonijah Graham et al. developed a totally biocompatible fiber-based TENG to harvest mechanical energy for medical care monitoring [129]. Positive (+ve) and negative (−ve) triboelectric fibers were prepared by electrospinning PVA and PCL. Triboelectric energy harvester and sensor (TEHS) devices can be used for medical care monitoring, and all TEHS devices have almost the same output performance (Figure 14d). Additionally, TEHS can be used to harvest mechanical energy and power wearable electrical equipment. Furthermore, TEHS is used in various health monitoring systems, demonstrating that TENG based on biocompatible electrospinning fibers has considerable potential in energy-gathering and health care monitoring applications.

##### TENG Based on Synthetic Degradable Polymers for Implantable Medical Devices

**Figure 15:**
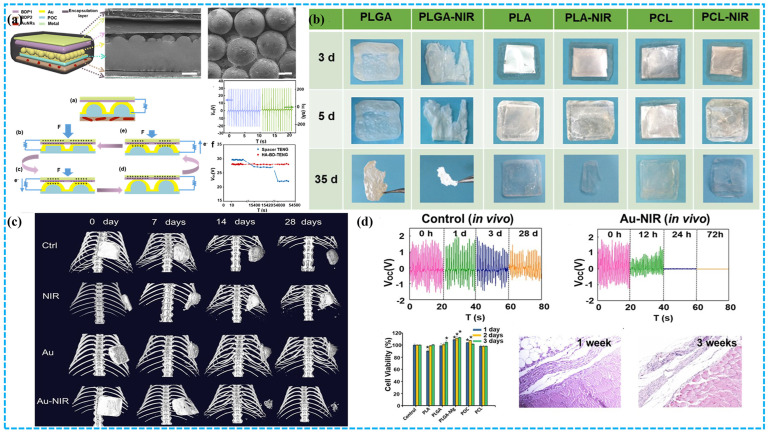
A solar-triggered transient energy harvester and sensor based on TENG using acid-sensitive polyphenylene glycol: (**a**) structure design of the photothermal-controlled BD-iTENG; working principle of the BD-iTENG. V_oc_ and I_sc_ of BD-iTENG; stability of spacer-based iTENG and as fabricated HA-iTENG; (**b**) biodegradation of the BD-iTENG made from PLGA, PLA and PCL in PBS at 37 °C with or without NIR laser; (**c**) micro-CT image of the implanted PLGA-iTENGs at various time points; (**d**) the electrical output of the PLGA-iTENG without AuNRs or NIR; (**c**) the electrical output of the PLGA-iTENG with AuNRs and NIR. Reproduced with permission [130], Copyright 2019, WILEY-VCH.

To adjust the degradation rate of the implanted equipment, Li et al. presented a biodegradable and implantable TENG (BD-iTENG) to develop a more capable and intelligent biological engineering [130]. They created a succession of triboelectric layers using Au-doped PLGA, PCL, and PLA. Near-infrared (NIR) photothermal treatments can initiate and regulate its deterioration performance (Figure 15). The V_oc_ and I_sc_ values of the BD-iTENG’s in vitro were 28 V and 220 nA, respectively. After implantation, the rats had a V_oc_ of 2 V. Moreover, BD-iTENG can operate successfully in vivo for more than 28 days without AuNRs doping and NIR treatments. After the NIR laser therapy, the device’s output voltage declined from 1.8 to 0.5 V in the 12th hour and further to 0 V in the body 24 h later. In vivo, the majority of the devices degraded after 14 days. It is reported that several attempts have controlled the working life of implanted biological TENG devices. However, further work is required to precisely adjust the degrading performance and reliability of implantable TENG devices.

The implantable power supply with controllable degradation is essential for the “transient” biomedical applications that will be utilized throughout the design era in the future. To satisfy these emerging demands, Zheng et al. developed a BD-TENG for the in-vivo harvesting of biomechanical energy that can be decomposed and reabsorbed at the end of its operation [46]. The output performance and degradation characteristics of an implantable TENG power supply based on BD-polymers (PHB/V, PLGA, PVA, and PCL) and resorbable m3etals (such as Mg) can be adjusted by using the above different BD-polymers as triboelectric materials. When BD-TENG is used to power two complimentary micro grid electrodes, a DC pulsed electric field is formed, and nerve cell development is effectively orientated, demonstrating its viability in the neuron healing process. Within three days, the devices can be entirely dissolved in rats.

## 4. Challenges and Opportunities

In recent years, due to the detailed research on materials and structure design, BD-TENG has shown great development potential and bright prospects in energy collection, self-powered sensing and implantable medicine. However, as a new research field, there are still some challenges to overcome. Here, we discussed the direction and future prospects of BD-TENG optimization in terms of components and performance.

### 4.1. Biodegradable Materials for BD-TENGs

Biodegradable materials are a vital component of BD-TENGs and deserve more discussion. Firstly, the characteristics and application of BD-TENG will be affected by the material structure and its properties. Animal-based materials generally contain rich nitrogen groups, so they have stronger electronegativity than some plant materials. Therefore, the surface potential of degradable materials can be altered by modification techniques like nitrification and amination, so as to limit the charge state during contact charging. Different components and modification techniques can enhance BD-TENG’s performance in varying degrees.

Secondly, biocompatibility and biodegradability are significantly influenced by a material’s molecular structure, which may affect how BD-TENG is applied in vivo or in vitro. For example, particular molecular locations, like peptide bonds can be quickly detected and broken down by organisms. On the other hand, some molecular configurations will endanger organisms’ ability to live normally. In this regard, the biocompatibility and biodegradability of materials will be affected by material alteration.

The surface microstructure has an important influence on the output of BD-TENG. The surface microstructure is affected by several material preparation and modification procedures which has been demonstrated. Specific surface microstructures can be successfully created using materials preparation techniques like water photolithography and electrospinning. To enhance the functionality of TENG, additional techniques like ICP etching and plasma cleaning can be used to create microstructures on the material surface.

It is worth noting that most BD-TENG reported at present are not completely degradable. Plant based degradable materials, animal based degradable materials and synthetic degradable materials are basically used for the positive friction electric layer of TENG, while the negative friction electric layer is still made of non-degradable polymers such as PTFE, FEP, PVDF, PDMS, etc. These TENGs are not completely degradable. Therefore, researchers should devote themselves to the research and development of fully biodegradable TENG in the future.

### 4.2. Output Performance of BD-TENG

The output performance of BD-TENG still needs to be improved. In Table 1, we summarized TENG’s recent research reports using degradable materials as the triboelectric layer and summarized the properties of these materials. There are few BD-TENGs with high output, which may hinder the clinical application of BD-TENG. Therefore, it is challenging to drive electronic devices or achieve effective electrical stimulation of BD-TENG. Particularly for human-integrated devices, the mechanical qualities of materials are crucial for BD-TENG-based devices. For instance, extensibility and flexibility will directly affect user comfort. In addition, considering the future pursuit of device functionality, more material modification techniques will be applied on BD-TENGs including doping and chemical adjustment. Other procedures like structural transformation can also efficiently improve the output of TENG in addition to material change.

### 4.3. Packaging Technology of BD-TENGs

There is a greater demand for efficient packaging of implantable or wearable TENG devices made of biodegradable materials. The rapid corrosion of biodegradable materials will reduce the life and stability of the equipment. The wearability and even the insertion of TENG depend on good packing. The current packaging materials, such as PDMS, epoxy resin, and poly(p-xylene), have poor physical toughness. Future construction of the BD-TENG needs a flexible, even elastic, packaging structure.

### 4.4. Controllable Degradability of BD-TENGs

Currently, only some components of the BD-TENGs are composed of biodegradable materials. In the long run, it is highly anticipated that all of the BD-TENGs can be fabricated with green materials with controllable biodegradability and low cost. Another challenge is the controlling of TENG’s degradation. If the degradation happens too quickly, the service time of the equipment could not satisfy the treatment requirements. Conversely, security problems may occur if the downgrade was too slow. It is reported that the decomposition of BD-TENG has been manipulated using methanol treatment, near-infrared control, and other materials. But it is impossible to control material degradation very precisely. There are still different methods to prevent degradation, including physical, biological, and pharmacological stimulation. Chances and difficulties always complement one another. In the future, green biodegradable materials will increasingly be used in daily life due to environmental pollution and global warming, and by combining biodegradable materials with TENGs efficiently, future advancements in green energy technology will be definitely facilitated.

### 4.5. Application of BD-TENG

BD-TENGs show a bright application prospect in environmental protection, human monitoring, and rehabilitation. In recent work, the combination of TENG with solar energy is considered to produce solar/triboelectric components. Besides, the biodegradable triboelectric electromagnetic hybrid nanogenerator has also demonstrated a high blue energy conversion efficiency. These discoveries have positively influenced the multifunctional integrated development of BD-TENGs. BD-TENGs investigated in additional fields should be encouraged to further accelerate their commercialization process and enhance user satisfaction.

## 5. Summary and Perspectives

TENG is recognized as an innovative energy conversion technology because it introduced a simple and novel approach for converting mechanical energy into electrical energy. Many TENGs have been recently developed based on biodegradable materials, including gelatin, chitosan, silk fibroin, cellulose, alginate, and others. TENGs based on such biodegradable materials give birth to self-powered systems with several more advantages, particularly in self-powered therapy and diagnostics. It can be concluded that BD-TENGs may further positively affect our daily life in the future, from environmentally friendly energy harvesting to biofriendly self-powered active sensing and implantable medical devices.

This review covers the progress of BD-TENGs in terms of operating mechanisms, material sources, and application situations. Biodegradable materials for TENGs are classified into three types: plant-based biodegradable polymers such as cellulose, leaves, wood, rice paper, and alginate; animal-based biodegradable polymers such as silk protein, chitosan, gelatin, egg white, and polypeptide compounds; and synthetic degradable polymers including PVA, PLA, PLGA, PCL, PHB/V, etc. We also discussed the attributes of these materials and the merits of incorporating them into TENG, which has boosted the next generation of renewable energy techniques for environmentally friendly energy harvesting, self-powered active sensing, and degradable implantable medical equipment.

We have already forecast the future development direction of BD-TENG. We believe that the next research should emphasize creating innovative biodegradable functional materials with quick preparation capabilities, superior triboelectric characteristics, controllable biodegradable parts, and biodegradability. A new generation of multifunctional, environmentally friendly, outstanding performance, and flexible BD-TENGs is expected to combine with environmentally beneficial materials and eventually phase out synthetic polymers and toxic elements, thus eventually ushering a new age for biodegradable energy-gathering technology.

## Figures and Tables

**Table 1:** Performance comparison of BD-TENGs with different triboelectric materials.

Degradable Material Type	Material	The Other Electrode	Biodegradable/Biocompatible	Electrical Output	Application	Ref.
Plant-based	CCP	NCM	Both	196.8 V/31.5 μA	Energy harvesting, self-powered active sensing	[68]
Plant-based	Tissue paper	PVC	Biodegradable	100 V	Energy harvesting	[40]
Plant-based	Recycled papers	PTFE	Both	205 V/18 μA	Self-powered active sensing system	[69]
Plant-based	Wheat straw	FEP	Biodegradable	250 V/12 μA	Energy harvesting	[70]
Plant-based	Nutshell powder	PTFE	Biodegradable	248.9 V/39.4 μA	Energy harvesting	[71]
Plant-based	Rice paper	PVC	Biodegradable	244 V/6 μA	Energy harvesting	[64]
Plant-based	Laver	FEP	Both	23 V/315 nA	Energy harvesting	[73]
Plant-based	Leaves	Water droplets	Biodegradable	1 V/4 μA	Energy harvesting	[74]
Plant-based	BC	Cu	Both	13 V	Energy harvesting	[75]
Plant-based	BC	BC-CNT-PPy	Both	29 V/0.6 μA	Energy harvesting	[79]
Plant-based	Soy protein and lignin	PI	Both	~5 V	Energy harvesting	[76]
Plant-based	Wood	Wood	Biodegradable	90.1 V/114.4 Na·cm^−2^	Energy harvesting	[77]
Plant-based	Modified cellulose	PTFE	Both	335 V, 9.74 μA	Energy harvesting	[78]
Plant-based	ϰC-Agar	PCL	Both	30 V/0.45 mA·m^−2^	Energy harvesting	[81]
Plant-based	Cu-coated CFP	PVDF coated CFP	Both	192 V/9.3 μA	Energy harvesting	[82]
Plant-based	Cellulose paper	NC membrane	Both	60 V/8.8 μA	Energy harvesting	[83]
Plant-based	CNFs	FEP	Biodegradable	~30 V/~90 μA	Energy harvesting	[84]
Plant-based	Leaf	PMMA	Biodegradable	230 V/9.5 μA	Energy harvesting	[85]
Plant-based	Leaf	PVDF	Biodegradable	1000 V/60 μA	Energy harvesting	[86]
Plant-based	CMFs/CNFs	FEP	Both	21.9 V/0.17 µA	Self-powered active sensing system	[88]
Plant-based	Wood	PTFE	Biodegradable	81 V/1.8 µA	Self-powered active sensing system	[89]
Plant-based	EC	317L SS	Both	245 V/50 µA	Implantable medical devices	[91]
Animal-based	Polypeptide	PTFE	Both	~350 V/10 μA	Energy harvesting	[96]
Animal-based	chitosan-diatom	FEP	Both	150 V/1.02 µA	Self-powered active sensing system	[103]
Animal-based	SF	PVA/MXene	Both	118 V	Self-powered active sensing system	[109]
Animal-based	SF	PTFE	Both	~50 V/~3 μA	Self-powered active sensing system	[110]
Animal-based	SF/PAM/GO	PDMS	Both	12 V/0.4 μA	Self-powered active sensing system	[111]
Animal-based	SNRF	RSFF	Both	41.64 V/0.5 μA	Self-powered active sensing system	[112]
Animal-based	SF	PDMS	Both	13 V/0.4 μA	Self-powered active sensing system	[101]
Animal-based	FG	PTFE/PDMS	Both	130 V, 0.35 μA	Self-powered active sensing system	[104]
Animal-based	FBF	Cu	Both	106 V/7.3 μA	Self-powered active sensing system	[113]
Animal-based	gelatin	PDMS	Both	11 V/3 mA m^−2^	Self-powered active sensing system	[102]
Animal-based	RSSP	PET	Both	2600 V/480 μA	Implantable medical devices	[115]
Animal-based	SF	Mg	Both	~60 V/~1 μA	Implantable medical devices	[39]
Animal-based	Silk/silk fibroin	Rice paper & cellulose	Both	~40 V/0.4 μA & ~25 V/0.3 μA	Implantable medical devices	[37]
Synthetic material	PLA/PLGA	Mg	Both	26 V, 108 nA	Energy harvesting	[126]
Synthetic material	PVA	PS	Both	30 V/~1 μA	Energy harvesting	[131]
Synthetic material	PLA	PVDF-HFP	Biodegradable	140 V/3.8 μA	Self-powered active sensing system	[127]
Synthetic material	PLA	gelatin	Both	500 V/10 mA m^−2^	Self-powered active sensing system	[108]
Synthetic material	PPHA	Kapton	Biodegradable	~36 V	Self-powered active sensing system	[128]
Synthetic material	PCL	PVA	Both	~8 V/~2.6 µA	Self-powered active sensing system	[129]
Synthetic material	PLGA/PCL/PLA	Au nanorods	Both	28 V/220 nA	Implantable medical devices	[130]
Synthetic material	PHB/V	PCL/PVA/PLGA	Both	28 V/0.6 μA, 13 V/0.2 μA, 15 V/0.3 μA	Implantable medical devices	[46]

## Data Availability

Data available on request from the authors.
